# Cartan’s incomplete classification and an explicit ambient metric of holonomy $\mathrm{G}_2^*$

**DOI:** 10.1007/s40879-017-0178-9

**Published:** 2017-09-12

**Authors:** Travis Willse

**Affiliations:** Fakultät für Mathematik, Universität Wien, Oskar-Morgenstern-Platz 1, 1090 Vienna, Austria

**Keywords:** (2, 3, 5) distributions, Conformal geometry, Exceptional holonomy, Fefferman–Graham ambient construction, $\mathrm{G}_2$, Generic distributions, Metric holonomy, 53A30, 53C29, 58A30

## Abstract

In his 1910 “Five Variables” paper, Cartan solved the equivalence problem for the geometry of (2, 3, 5) distributions and in doing so demonstrated an intimate link between this geometry and the exceptional simple Lie groups of type $\mathrm{G}_2$. He claimed to produce a local classification of all such (complex) distributions which have infinitesimal symmetry algebra of dimension at least 6 (and which satisfy a natural uniformity condition), but in 2013 Doubrov and Govorov showed that this classification misses a particular distribution $\mathbf {E}$. We produce a closed form for the Fefferman–Graham ambient metric ${\smash {{\smash {\widetilde{g}}}}}_{\mathbf {E}}$ of the conformal class induced by (a real form of) $\mathbf {E}$, expanding the small catalogue of known explicit, closed-form ambient metrics. We show that the holonomy group of $\smash {{\smash {\widetilde{g}}}_{\mathbf {E}}}$ is the exceptional group ${\smash {\mathrm{G}}_2^*}$ and use that metric to give explicitly a projective structure with normal projective holonomy equal to that group. We also present some simple but apparently novel observations about ambient metrics of general left-invariant conformal structures that were used in the determination of the explicit formula for $\smash {{\smash {\widetilde{g}}}_{\mathbf {E}}}$.

## Introduction

Cartan’s most involved application of his powerful method of equivalence was the resolution of the (local) equivalence problem for the geometry of 2-plane distributions $\mathbf {D}$ on 5-manifolds *M*, which he reported in his landmark 1910 “Five Variables” paper [14]. Such distributions that are maximally nonintegrable in the sense thatare called *(2, 3, 5) distributions* because 2, 3, 5 are the respective ranks of the vector bundles in the derived filtration . This geometry has proved interesting for numerous reasons: It comprises a first class of distributions with continuous local invariants, it is connected to the geometry of surfaces rolling on one another without slipping or twisting [5, 8, 10] and hence to natural problems in control theory [2], it is intimately linked (as Cartan showed in the aforementioned article^1^) to the exceptional simple complex Lie group of type $\mathrm{G}_2^{\mathbb {C}}$ and then via the natural inclusion  to conformal geometry [26], and it is the appropriate structure for naturally projectively compactifying strictly nearly (para-)Kähler structures in dimension 6 [21]. (Here, $\mathrm{G}_2^*$ denotes the automorphism group of the algebra of split octonions, one of the split real forms of the complex, connected simple Lie group $\mathrm{G}_2^{\mathbb {C}}$.) In turn, via the Fefferman–Graham ambient construction [18] and a general extension theorem [22] these structures can be used to construct metrics of holonomy contained in and sometimes equal to $\mathrm{G}_2^*$.

Cartan claimed to classify locally all (implicitly, complex) (2, 3, 5) distributions $(M, \mathbf {D})$ that both (a) have infinitesimal symmetry algebra  of dimension at least 6 and (b) satisfy a natural uniformity condition called *constant root type* (see Sect. 3). The algebra  consists of all vector fields  whose flow preserves $\mathbf {D}$, or equivalently, for which $\mathscr {L}_X Y \in \mathrm{\Gamma }(\mathbf {D})$ for all $Y \in \mathrm{\Gamma }(\mathbf {D})$. Some 103 years later, Doubrov and Govorov upended this classification [16] by showing that it misses a distribution, which we denote $\mathbf {E}$, and which turns out to be a left-invariant distribution on a particular Lie group. In fact, Doubrov and Govorov reported finding this missing distribution to the author during his preparation of an earlier article [30] that relied on Cartan’s classification; this distribution was discussed briefly in Example 38 and Remark 39 in that article, but detailed discussion was deferred to the present article.

Nurowski showed that any (2, 3, 5) distribution $(M, \mathbf {D})$ canonically induces a conformal structure $\mathbf {c}_{\mathbf {D}}$ of signature (2, 3) on *M* [26], and he later showed with Leistner that one can exploit certain distributions of this type to produce explicit *ambient metrics* ${\smash {\widetilde{g}}}$ with metric holonomy  equal to $\mathrm{G}_2^*$. The ambient metric construction assigns to a conformal structure $(M, \mathbf {c})$ of signature (*p*, *q*) an essentially unique Riemannian metric $({\smash {\widetilde{M}}}, {\smash {\widetilde{g}}})$ of signature ; see Sect. 4.1. Though the ambient metric construction enjoys a satisfying existence theorem, producing an explicit ambient metric for a particular conformal structure amounts to solving a formidable nonlinear system of partial differential equations on *M*, and consequently explicit closed forms for ambient metrics are known only for a few special classes of conformal structures [6], [18, Section 3],  [20, Theorem 2.1], [24].

We observe in Sect. 4.2 that in the special case that $\mathbf {c}$ is a left-invariant conformal structure on a Lie group, after making certain natural choices the system becomes one of *ordinary* differential equations. In particular this applies to the left-invariant conformal structure $\mathbf {c}_{\mathbf {E}}$ determined by $\mathbf {E}$; here we abuse notation by using the same notation $\mathbf {E}$ for a particular real distribution, as well as Doubrov and Govorov’s missing complex distribution, which is in a sense that can be made precise a complexification of that real distribution. In Sect. 5 we manage to solve explicitly the system of ordinary differential equations determined by $\mathbf {c}_{\mathbf {E}}$ and hence produce for it an explicit ambient metric ${\smash {\widetilde{g}}}_{\mathbf {E}}$, and its behavior turns out to be qualitatively different from previous explicit examples; see Remark 5.2. Furthermore, computing using the Ambrose–Singer Theorem gives that the holonomy group of ${\smash {\widetilde{g}}}_{\mathbf {E}}$ is $\mathrm{G}_2^*$, furnishing a new example a metric with that exceptional holonomy group. Finally, we apply some general facts relating ambient metrics to projective structures whose normal tractor connections enjoy orthogonal holonomy reductions [21] to produce from ${\smash {\widetilde{g}}}_{\mathbf {E}}$ an explicit example of a projective structure with normal projective holonomy $\mathrm{G}_2^*$.

Many computations whose results are reported here were carried out with Ian Anderson’s Maple Package DifferentialGeometry.

## (2, 3, 5) distributions

Given two distributions  on a (real or complex) manifold, their bracket $[\mathbf {D}, \mathbf {D}']$ is the set . (A priori this set need not be a distribution, as the rank of the vector space  may vary with *u*.) Short computations give that for a distribution $\mathbf {D}$ we have , and that if  is a distribution, then . (Implicit in the notation  is the requirement that  itself be a distribution.)

For reasons including those mentioned in the introduction, the class of distributions that the current article concerns is especially interesting:

### Definition 2.1

A *(2, 3, 5) distribution* 2 is a 2-plane distribution $\mathbf {D}$ on a (real or complex) 5-manifold *M* that satisfies the genericity conditionTwo such distributions $(M, \mathbf {D})$ and  are *equivalent* iff there is a diffeomorphism $\phi :M \rightarrow M'$ such that . They are *locally equivalent* (near $u \in M$ and ) if there are neighborhoods *U* of *u* and $U'$ of $u'$ such that $(U, \mathbf {D}|_U)$ and  are equivalent via a diffeomorphism that maps *u* to $u'$.

The geometry of these structures was first studied systematically by Cartan, in his well-known “Five Variables” article [14]. There, he solved the equivalence problem for this geometry by canonically assigning to each such distribution $(M, \mathbf {D})$ a principal *Q*-bundle $E \rightarrow M$ and a $\mathfrak {g}_2^*$-valued pseudoconnection $\omega$ on *E*, where *Q* is a particular parabolic subgroup of $\mathrm{G}_2^*$.^3^

We here consider (2, 3, 5) distributions whose *infinitesimal symmetry algebra* is large:

### Definition 2.2

A vector field $X \in \mathrm{\Gamma }(TM)$ is an *infinitesimal symmetry* of a distribution $(M, \mathbf {D})$ iff it preserves $\mathbf {D}$ in the sense that $\mathscr {L}_X Y \in \mathrm{\Gamma }(\mathbf {D})$ for all $Y \in \mathrm{\Gamma }(\mathbf {D})$. The Lie algebra  of all such fields is the *infinitesimal symmetry algebra* of $(M, \mathbf {D})$.

### Monge (quasi)normal form

This geometry admits a local quasinormal form: Any ordinary differential equation1can be regarded as a differential system on the partial jet space ), where the variables *p* and *q* are placeholders respectively for $y'$ and $y''$, comprising the canonical forms , and the particular form . These forms are linearly independent at each point, so their common kernel $\mathbf {D}_F$ is a 2-plane distribution on , namely,Concretely, a pair (*y*(*x*), *z*(*x*)) is a solution of the equation (1) iff its prolongation  is an integral curve of $\mathbf {D}_F$. Computing directly shows that $\mathbf {D}_F$ is a (2, 3, 5) distribution iff the second derivative $F_{qq}$ vanishes nowhere.

Goursat showed that every (2, 3, 5) distribution is locally equivalent (around any point) to $\mathbf {D}_F$ for some *F* [19, Section 76]. A distribution $\mathbf {D}_F$ specified by a function *F* is said to be in *Monge (quasi)normal form*.

### The canonical conformal structure

Nurowski showed that any real (2, 3, 5) distribution $(M, \mathbf {D})$ determines a canonical conformal structure $\mathbf {c}_{\mathbf {D}}$ on the underlying manifold *M* [26, Section 5.3], and the argument in that reference shows that Nurowski’s construction applies just as well to establish that a complex (2, 3, 5) distribution induces a complex conformal structure on the underlying manifold.

Nurowski also gave an explicit (and, with a length of about 60 terms, daunting) formula [26, (54)] for a representative of the conformal structure $\mathbf {c}_{\mathbf {D}_F}$ of a distribution $\mathbf {D}_F$ in Monge normal form; its coëfficients in the coördinate coframe are polynomials of degree 6 and lower in the 4-jet of *F*.

## A missing distribution

### Cartan’s ostensible classification

In [14] Cartan claimed to classify, up to local equivalence, and implicitly in the complex setting, all (2, 3, 5) distributions whose infinitesimal symmetry algebra has dimension at least 6 and which have constant *root type*: The fundamental curvature invariant of a (2, 3, 5) distribution $(M, \mathbf {D})$—analogous to the Riemann curvature tensor in Riemannian geometry—is a section $A \in \mathrm{\Gamma }(\bigodot ^4 \mathbf {D}^*)$. The value of *A* at $u \in M$ depends on the 4-jet of $\mathbf {D}$ at *u*, and the quantity *A* is natural in the sense that it is preserved by diffeomorphism. The *root type* of $\mathbf {D}$ at $x \in M$ is just the collection of multiplicities of the zeros of $A_x$ viewed as a polynomial on the projective line $\mathbb {P}(\mathbf {D})$, or if $\mathbf {D}$ is real, on .^4^ A distribution has *constant root type* if the root type is the same for every point, and of course, homogeneous (2, 3, 5) distributions have this property.

The structures he found were the following, organized by root type. (The section numbers indicate the corresponding (sub)sections in [14].)

- (Section 8: *A* identically zero) The *flat model*  of the geometry, which can be realized locally (a) via the algebra of the split octonions [28], (b) as the so-called *rolling distribution* for two spheres whose radii have ratio  (see the item for Section 50 below for references), and (c) as the distribution given in Monge normal form  [26]; .
- (Section 9: *A* has a single quadruple root at each point) A class of distributions $\mathbf {D}_I$ specified by a single function *I* of one variable. If *I* is constant then $\mathbf {D}_I$ is homogeneous and ; otherwise  and $\mathbf {D}_I$ is not homogeneous. In both cases  is solvable.
- (Section 11: *A* has two double roots at each point) A family of distributions parameterized by one complex parameter. Up to isomorphism, three different infinitesimal symmetry algebras occur, all of which have dimension 6.
  - (Section 50) A general distribution in the family has infinitesimal symmetry algebra isomorphic to . This family includes appropriate complexifications of the rolling distributions of two real surfaces of different nonzero constant curvature whose curvatures do not have ratio ; for that ratio the rolling distribution is flat. See [1, 7, 8, 10, 32] for much more.
  - (Section 51) There is a single distribution with infinitesimal symmetry algebra isomorphic to .
  - (Section 52) There is a single distribution with infinitesimal symmetry algebra isomorphic to . This distribution is locally equivalent to the appropriate complexification of the rolling distribution of a real surface of constant curvature and a plane.

### Doubrov–Govorov’s distribution

Some 103 years after Cartan’s work, Doubrov and Govorov found a complex (2, 3, 5) distribution $\mathbf {E}$ that has 6-dimensional infinitesimal symmetry algebra but which is missing from Cartan’s classification.

Define a Lie algebra structure $\mathfrak {h}$ on  ($\mathbb {F}= \mathbb {R}$ or $\mathbb {F}= \mathbb {C}$) by the bracket multiplication tablewhere $(E_a)$ is any basis, and define . Then, let *H* be a connected Lie group with Lie algebra $\mathfrak {h}$, regard $\mathbf {E}_0$ as a subspace of $T_{\mathrm{id}} H$ via its identification with $\mathfrak {h}$, and let  be the left-invariant 2-plane distribution on *H* characterized by . Via a usual abuse of notation, let $(E_a)$ also denote the left-invariant frame on *H* whose restriction to $\mathrm{id}\in H$ is the basis $(E_a) \subset \mathfrak {h}\cong T_{\mathrm{id}} H$; then, computing using the above bracket relations gives  , and so $\mathbf {E}$ is a (2, 3, 5) distribution.

Doubrov and Govorov integrated these structure equations and produced a Monge normal form for the distribution, given by the function , on a suitable domain. For readability, we use coördinates , where $q = r^3$.

One can verify directly that $F_{\mathbf {E}}$ defines a Monge normal form for $\mathbf {E}$ by showing that the frame $L_a$ defined bysatisfies the bracket relations of $(E_a)$ and that the identifications $E_a \leftrightarrow L_a$ identify $\mathbf {E}$ with the distribution determined by $F_{\mathbf {E}}$ (we henceforth make these identifications locally).

Since $\mathbf {E}$ is left-invariant, its infinitesimal symmetry algebra  contains the Lie algebra of right-invariant vector fields on *H*; one basis of this subalgebra isDirect computation shows that the vector field , which is not right-invariant, is also in . Tedious verification shows that this field and the right-invariant fields together span the infinitesimal symmetry algebra and establishes that , where $\mathfrak {n}_3$ is the 3-dimensional Heisenberg algebra over $\mathbb {F}$, and hence .

#### Proposition 3.1

([16]) The (complex) homogeneous (2, 3, 5) distribution $(H, \mathbf {E})$ is not locally equivalent to any of the distributions in Cartan’s list. In particular, Cartan’s list is incomplete.

#### Proof

Computing gives that the fundamental curvature of $\mathbf {E}$ is $A_{\mathbf {E}} = (e^4)^4 \vert _{\mathbf {E}}/4$ (here, $(e^a)$ is the left-invariant coframe on *H* dual to $(E_a)$), so at each point it has a quadruple root at $[E_5] \in \mathbb {P}(\mathbf {E})$. But there is no homogeneous distribution $\mathbf {D}$ in Cartan’s list with this (constant) root type for which , so $\mathbf {E}$ is not equivalent to any distribution on that list. $\square$

#### Remark 3.2

One can establish Proposition 3.1 without the work of verifying that  is not larger than 6 by observing that all of the distributions $\mathbf {D}$ in Cartan’s list for which $A_{\mathbf {D}}$ has a quadruple root at every point have solvable infinitesimal symmetry algebras, whereas  contains the simple subalgebra  and so is not solvable.

## Ambient metrics

The Fefferman–Graham ambient construction associates to a conformal structure $(M, \mathbf {c})$ of signature (*p*, *q*), $\dim M = p + q \geqslant 2$, a unique pseudo-Riemannian metric $({\smash {\widetilde{M}}}, {\smash {\widetilde{g}}})$ of signature . When $\dim M$ is odd, this construction is essentially unique, and hence invariants of ${\smash {\widetilde{g}}}$ (that are independent of the choices made) are also invariants of the underlying conformal structure; indeed, this was the original motivation for the construction [18].

### The Fefferman–Graham ambient metric construction

We describe the ambient metric construction for odd dimension $n > 1$ following [18].

Fix a conformal structure $\mathbf {c}$ of dimension *n*. The *metric bundle* associated to $\mathbf {c}$ is the ray bundle $\pi :\mathscr {G}\rightarrow M$ defined byThe bundle $\mathscr {G}$ enjoys a natural topology and smooth structure such that its smooth sections are precisely the representative metrics of $\mathbf {c}$. The natural dilation action  realizes $\mathscr {G}$ as a principal $\mathbb {R}_+$-bundle. Also, $\mathscr {G}$ admits a tautological symmetric 2-tensor  defined by
$\mathbf {g}_0$ annihilates $\pi$-vertical vectors and hence is degenerate. By construction, $\delta _s^* \mathbf {g}_0 = s^2 \mathbf {g}_0$.

Fixing a representative $g \in \mathbf {c}$ trivializes $\mathscr {G}\leftrightarrow \mathbb {R}_+ {\times } M$ via the identification $t^2 g_u \leftrightarrow (t, u)$. With respect to this trivialization, the tautological 2-tensor is given by .

Now, consider the space  and denote the standard coördinate on $\mathbb {R}$ by $\rho$. Then, the map  defined by $z \mapsto (z, 0)$ embeds $\mathscr {G}$ as a hypersurface in  and we identify $\mathscr {G}$ with this hypersurface. The dilations $\delta _s$ extend trivially to , that is, by . Likewise, a choice of representative $g \in \mathbf {c}$ determines a trivialization  that identifies , which in turn defines an embedding  and yields an identification . As is conventional, we denote indices corresponding to the factor $\mathbb {R}_+$ by 0, those corresponding to *M* by lowercase Latin letters, $a, b, c, \ldots$, and those corresponding to the factor $\mathbb {R}$ by $\infty$.

A smooth metric ${\smash {\widetilde{g}}}$ of signature  on an open neighborhood ${\smash {\widetilde{M}}}$ of $\mathscr {G}$ in  invariant under the dilations $\delta _s$, $s \in \mathbb {R}_+$, is a *pre-ambient metric* for $(M, \mathbf {c})$ if

- it extends $\mathbf {g}_0$ in the sense that $\iota ^* {\smash {\widetilde{g}}}= \mathbf {g}_0$, and
- it is homogeneous of degree 2 with respect to the dilations $\delta _s$, that is, $\delta _s^* {\smash {\widetilde{g}}}= s^2 {\smash {\widetilde{g}}}$.

A pre-ambient metric is *straight* if for all $u \in {\smash {\widetilde{M}}}$ the parameterized curve $\mathbb {R}_+ \rightarrow {\smash {\widetilde{M}}}$ defined by $s \mapsto s {\cdot }u$ is a geodesic. Any (nonempty) conformal structure admits many pre-ambient metrics; Cartan’s normalization condition for a conformal connection [15] suggests that Ricci-flatness is a natural distinguishing criterion.

#### Definition 4.1

Let $(M, \mathbf {c})$ be a conformal manifold of odd dimension at least 3. An *ambient metric* for $(M, \mathbf {c})$ is a straight pre-ambient metric ${\smash {\widetilde{g}}}$ for $(M, \mathbf {c})$ such that the Ricci curvature ${\smash {\widetilde{R}}}$ of ${\smash {\widetilde{g}}}$ is $O(\rho ^{\infty })$; the pair $({\smash {\widetilde{M}}}, {\smash {\widetilde{g}}})$ is an *ambient manifold* for $(M, \mathbf {c})$.

Here, we say that a tensor field on ${\smash {\widetilde{M}}}$ is $O(\rho ^{\infty })$ if it vanishes to infinite order in $\rho$ at each point in the zero set $\mathscr {G}$ of $\rho$.

We formulate Fefferman–Graham’s existence and uniqueness results for ambient metrics of odd-dimensional conformal structures as follows:

#### Theorem 4.2

([18]) Let $(M, \mathbf {c})$ be a conformal manifold of odd dimension at least 3. There is an ambient metric for $(M, \mathbf {c})$, and it is unique up to infinite order: If ${\smash {\widetilde{g}}}_1$ and ${\smash {\widetilde{g}}}_2$ are ambient metrics for $(M, \mathbf {c})$, then (after possibly restricting the domains of both to appropriate open neighborhoods of $\mathscr {G}$ in ${\smash {\widetilde{M}}}$) there is a diffeomorphism $\mathrm{\Phi }$ such that $\mathrm{\Phi }\vert _{\mathscr {G}} = \mathrm{id}_{\mathscr {G}}$ and $\mathrm{\Phi }^* {\smash {\widetilde{g}}}_2 - {\smash {\widetilde{g}}}_1$ is $O(\rho ^{\infty })$.

The proof of the theorem uses the fact that for any representative $g \in \mathbf {c}$ we may write any ambient metric ${\smash {\widetilde{g}}}$ (possibly after restricting to an open, dilation-invariant set containing $\mathscr {G}$) in the *normal form*
2where  and where we may regard  as a family of metrics on *M* depending on the parameter $\rho$; here and henceforth we suppress notation for pullback by the inclusion $M \hookrightarrow {\smash {\widetilde{M}}}$ determined by *g*.

We want to write the Ricci-flatness condition ${\smash {\widetilde{R}}}= 0$ for a metric ${\smash {\widetilde{g}}}$ on ${\smash {\widetilde{M}}}$ in normal form: The components ${\smash {\widetilde{R}}}_{00}, {\smash {\widetilde{R}}}_{0a}, {\smash {\widetilde{R}}}_{0\infty }$ of  with respect to the splitting  are zero. The vanishing of the remaining components ${\smash {\widetilde{R}}}_{ab}, {\smash {\widetilde{R}}}_{a\infty }, {\smash {\widetilde{R}}}_{\infty \infty }$ is equivalent to a system of $(n + 2)(n + 1)/2$ partial differential equations in $g_{ab}$; these components are given by [18, (3.17)]3here , $'$ denotes differentiation with respect to $\rho$, and $\nabla _a$ and $R_{ab}$ respectively denote the Levi-Civita connection and Ricci curvature of  with $\rho$ fixed.

Differentiating these expressions, setting them equal to zero, and then evaluating at $\rho = 0$ successively determines all of the derivatives . If the representative *g* is real-analytic, then the Taylor series of  about $\rho = 0$ (that is, along $\mathscr {G}$) converges to a real-analytic ambient metric ${\smash {\widetilde{g}}}$ on some open subset of ${\smash {\widetilde{M}}}$ containing $\mathscr {G}$, and in particular ${\smash {\widetilde{\mathrm{Ric}}}}= 0$.

Despite the existence of ambient metrics that Theorem 4.2 guarantees, explicit examples have been produced for only a few isolated classes of conformal structures [18, Section 3], [20, Theorem 2.1], [24], [23, Section 2], owing in part to the severe nonlinearity of the system ${\smash {\widetilde{\mathrm{Ric}}}}= 0$. Nurowski produced explicit ambient metrics for the conformal structures induced by a special class of (2, 3, 5) distributions [27] and with Leistner showed that most of these metrics have holonomy $\mathrm{G}_2^*$ [25]; the same has been accomplished for a proper superset of this family, namely for the conformal structures induced by the distributions given in Monge normal form by the functions  [6].

### Ambient metrics of left-invariant conformal structures

The typically intractable problem of computing explicit ambient metrics of particular conformal structures in principle simplifies significantly (but in general remains difficult) in the special case of left-invariant conformal structures on Lie groups. We indicate some of these simplifications here and treat this problem in more detail in a work in progress [31].

Given a Lie group *G*, let $L_h :G \rightarrow G$ be the left multiplication map $k \mapsto hk$. A conformal structure $\mathbf {c}$ on a Lie group *G* is *left-invariant* iff $L_h^* \mathbf {c}= \mathbf {c}$ for all $h \in G$, that is, iff for any (equivalently, every) representative metric $g \in \mathbf {c}$ we have $L_h^* g \in \mathbf {c}$. Any such conformal structure contains a distinguished 1-parameter family of representative metrics, namely the left-invariant ones: Any  determines a unique left-invariant metric *g* satisfying , and by left-invariance  for all $h \in G$, that is, $g \in \mathbf {c}$. Since the choice of  is arbitrary, all left-invariant metrics in $\mathbf {c}$ arise this way.

The problem of finding an explicit expression for the ambient metric simplifies in a critical way when writing the ambient metric in normal form (2) with respect to a left-invariant representative metric *g*.

#### Proposition 4.3

Let $(G, \mathbf {c})$ be a left-invariant conformal structure on a Lie group of odd dimension $n \geqslant 3$ and $g \in \mathbf {c}$ a left-invariant representative metric, and let $(E_a)$ be any left-invariant frame on *G*.

(i) There is a real-analytic—and hence Ricci-flat—ambient metric ${\smash {\widetilde{g}}}$ for $\mathbf {c}$ invariant under the trivial extension of the left action of *G* to the ambient space . In particular, for fixed $\rho$ the quantity  in the normal form (2) is a left-invariant metric on *G*, and so the components of  with respect to the left-invariant frame $(E_a)$ are functions $g_{ab}(\rho )$ of $\rho$ alone.
(ii) The components of the ambient metric system ${\smash {\widetilde{\mathrm{Ric}}}}= 0$ with respect to the (*G*-invariant) frame  of $T{\smash {\widetilde{G}}}$ comprise a system of ordinary differential equations in $g_{ab}(\rho )$.

Theorem 4.2 thus guarantees that any ambient metric for a left-invariant conformal structure is, informally, “invariant to infinite order” under the *G*-action on $\mathscr {G}$ specified therein.

#### Proof

(i) Since *g* is left-invariant it is real-analytic, and hence its local invariants are left-invariant and real-analytic, too, including its Levi-Civita connection $\nabla$, its Ricci curvature $\mathrm{Ric}$, and the covariant derivatives $\nabla ^k \mathrm{Ric}$ thereof. On the other hand, [18, Proposition 3.5] gives that for an ambient metric in normal form (2) each of the derivatives  of $g(u, \rho )$ is a certain linear combination of contractions of Ricci curvature and its covariant derivatives, and so in particular these derivatives are left-invariant and real-analytic. Thus, so is the real-analytic function  they define, and hence substituting this function in (2) yields a real-analytic ambient metric ${\smash {\widetilde{g}}}$ with the specified invariance property. By left-invariance of , so $g_{ab}(h, \rho )$ does not depend on *h* and hence we may write it as a function $g_{ab}(\rho )$ of $\rho$ alone.

(ii) The condition ${\smash {\widetilde{\mathrm{Ric}}}}= 0$ is equivalent to the vanishing of ${\smash {\widetilde{R}}}_{ab}$, ${\smash {\widetilde{R}}}_{a\infty }$, and ${\smash {\widetilde{R}}}_{\infty \infty }$, so its suffices to express each of those as differential expressions in $g_{ab}(\rho )$ involving only derivatives with respect to $\rho$. Note that the expression for $R_{\infty \infty }$ in (3) already has this form.

The only term in the expression for ${\smash {\widetilde{R}}}_{ab}$ in (3) that depends on derivatives of *g* in directions other than $\rho$ is $R_{ab}$, the Ricci curvature. Let $C_{ab}^c$ be the structure constants of *G* with respect to the frame $(E_a)$, that is, the unique constants that satisfy $[E_a, E_b] = C_{ab}^c E_c$ for all *a*, *b*, *c*; by the Koszul formula, the Levi-Civita connection $\nabla$ of a left-invariant metric *g* on *G* is given by4We can use this formula to express the Riemannian curvature tensor $R_{abcd}$ and hence the Ricci curvature $R_{ab}$ as a sum of formal contractions of the metric $g_{ab}$, its inverse, $g^{ab}$, and the structure constants, $C_{ab}^c$ of *G*. (The formula is unwieldy so we do not reproduce it here.)

By computing directly using (4) again along with the formula for the Levi-Civita connection ${\smash {\widetilde{\nabla }}}$ of an ambient metric in normal form (2), we can express the component , as


#### Remark 4.4

There is an even-dimensional analogues of Theorem 4.2, but it is more subtle: In short, for a conformal manifold of even dimension *n*, both formal existence and uniqueness of ambient metrics are guaranteed roughly only to order *n* / 2 in $\rho$ [18, Section 3]. There is a corresponding even-dimensional version of Proposition 4.3 but we delay its precise statement to [31].

#### Remark 4.5

Any left-invariant bilinear form on *G* is determined by its restriction to $T_{\mathrm{id}} G$, which we may identify with the Lie algebra $\mathfrak {g}$ of *G*. Thus, we can regard the ambient metric system ${\smash {\widetilde{\mathrm{Ric}}}}= 0$ as an ordinary differential equation on .

#### Remark 4.6

Proposition 4.3 and the following remarks hold just as well if one replaces *left-invariant* with *right-invariant* in all instances.

## A new explicit ambient metric

With some effort, one can produce an explicit ambient metric for the conformal structure $(H, \mathbf {c}_{\mathbf {E}})$. Let $(e^a)$ denote the left-invariant coframe on *H* dual to the frame $(E_a)$ defined in Sect. 3.2. Then, $\mathbf {c}_{\mathbf {E}}$ has the representative5


### Proposition 5.1

The metric6on  is an ambient metric for the (real) left-invariant conformal structure $(H, \mathbf {c}_{\mathbf {E}})$ induced by the (real) distribution $\mathbf {E}$.

### Proof

Evaluating the quantity in parentheses at $\rho = 0$ shows that ${\smash {\widetilde{g}}}_{\mathbf {E}}$ is in normal form with respect to $g_{\mathbf {E}}$, and computing directly shows that . $\square$

Since solving the system (3) outright is typically difficult, even for left-invariant conformal structures, we indicate briefly a naïve method for producing the explicit solution (6 for this particular case. First, note that for our chosen left-invariant frame $(E_a)$ most of the structure constants $C_{ab}^c$ (five of the 50 with $a < b$) are zero, as are most of the coefficients of the representative metric $g_{\mathbf {E}}$ and Ricci curvature7After computing the first several derivatives $\smash {g_{ab}^{(m)}(0)}$ of $g_{ab}(\rho )$ using the algorithm described after (3) some visible patterns emerge. First, only certain components $g_{ab}(\rho )$ have nonzero derivatives among them: Up to the symmetry $g_{ab} = g_{ba}$, only $g_{11}, g_{13}$, $g_{22}, g_{25}, g_{33}, g_{34}$ are nonzero.^5^ Second, the truncated Taylor series at $\rho = 0$ of $g_{25}, g_{34}$ agree. Third, the truncated Taylor series at $\rho = 0$ of the coëfficients $g_{11}(\rho )$, $g_{13}(\rho ), g_{22}(\rho ), g_{33}(\rho )$ coincide with those of simple rational functions, namely, those in (6).

These observations suggest the ansatzfor the quantity  in the normal form (2) for the ambient metric ${\smash {\widetilde{g}}}$ of $\mathbf {c}_{\mathbf {E}}$. Then, decomposing the equation  with respect to the frame  yields a system of ordinary differential equations in $a(\rho )$. The full system, which has eight nonzero components, is too large to reproduce here, but, e.g., the component $\mathrm{Ric}^{{\smash {\widetilde{g}}}}(E_2, \partial _{\rho }) = 0$ is a relatively simple first-order equation:This is separable and so can be solved readily by hand, and using the initial value $h(0) = g_{25}(0) = g_{34}(0) = 2$ gives the candidate solution $a(\rho ) = \sqrt{2 (2 + \rho )}$. Substituting shows that this candidate is a solution to the system, so the resulting metric ${\smash {\widetilde{g}}}_{\mathbf {E}}$ is indeed an ambient metric for $\mathbf {c}_{\mathbf {E}}$.

### Remark 5.2

This is a first example of an explicit, closed-form ambient metric not polynomial in the ambient coördinate $\rho$. Since this article was first uploaded to the arXiv, more examples were produced in [6], including some of the ambient metrics ${\smash {\widetilde{g}}}_{f, h}$ of conformal structures induced by (2, 3, 5) distributions  given respectively in Monge normal form by the functions . As an anonymous referee observed, a priori the conformal structure $\mathbf {c}_{\mathbf {E}}$ may be (locally) equivalent to the conformal structure $\mathbf {c}_{f, h}$ induced by a distribution  for some *f*, *h*—and hence ${\smash {\widetilde{g}}}_{\mathbf {E}}$ may occur (at least up to local isometry) in the family ${\smash {\widetilde{g}}}_{f, h}$—but it turns out this is not the case. We give a proof of this assertion here, in part because the techniques used may be of independent interest.

Suppose there are such *f*, *h*. We first show briefly that local equivalence of $\mathbf {c}_{\mathbf {E}}$ and $\mathbf {c}_{f, h}$ implies local equivalence of $\mathbf {E}$ and : Theorem C of [29] states that if for two different (2, 3, 5) distributions $\mathbf {D}, \mathbf {D}'$ the induced conformal structures satisfy  then that conformal structure admits a so-called almost Einstein scale (see Section 2.5 of that reference), in which case Proposition A of that reference implies that the normal conformal holonomy of that conformal structure is a proper subgroup of $\mathrm{G}_2^*$. On the other hand, Proposition 5.4 below shows that the metric holonomy  is equal to $\mathrm{G}_2^*$, and by [11, Corollary 1.2] it follows that the conformal holonomy  of $\mathbf {c}_{\mathbf {E}}$ is the full group $\mathrm{G}_2^*$, so $\mathbf {c}_{\mathbf {E}}$ does not admit an almost Einstein scale; hence $\mathbf {E}$ and  are locally equivalent.

Now, as observed in the proof of Proposition 3.1, the fundamental curvature $A_{\mathbf {E}}$ of $\mathbf {E}$ has a quadruple root at all points, and hence so does the fundamental curvature $A_{f, h}$ of $\mathbf {D}_{f, h}$, which computing (using the algorithm in [22, Section 5]) gives isfor a particular function *G*. In particular, since  has a quadruple root at all point, *f*, *h* must together satisfy , and differentiating both sides of this equation by *p* gives that *f* satisfies . The explicit formulae in [6, (3.5), Theorem 3.2] show that this latter condition implies that there is a representative metric $g_{f, h} \in \mathbf {c}_{f, h}$ for which the ambient metric in normal form with respect to $g_{f, h}$ is linear in $\rho$.

We can thus establish the claim by showing there is no $g \in \mathbf {c}_{\mathbf {E}}$ for which the ambient metric for $\mathbf {c}_{\mathbf {E}}$ in normal form with respect to *g* is linear in $\rho$.

For a general conformal structure $\mathbf {c}$ and choice $g \in \mathbf {c}$ of representative metric consider the Taylor series expansionabout $\rho = 0$ of the quantity  in (2). The first equation in [18, (3.18)] gives that (in dimension 5) the coëfficient $\smash {\mu ^{(2)}_{ab}}$ of the quadratic term is $-B_{ab} + P_a{}^c P_{bc}$, where (again specializing to dimension 5) *P* is the Schouten tensor of *g*, $P_{ab} = (R_{ab} - g^{cd} R_{cd} g_{ab}/8)/3$, *B* is the Bach tensor of *g*, $B_{ab} = P_{b[c, a]}{}^c + P^{cd} W_{cabd}$, and *W* is the Weyl tensor of *g*. Using the conformal invariance of *W* and the conformal transformation laws for *B* and *P* [18, pp. 56, 71, resp.] thus yields a formula for the coëfficient $\widehat{\mu }^{(2)}$ of the Taylor series expansion of the quantity  in (2) for an ambient metric in normal form with respect to the general representative $e^{2 \mathrm{\Upsilon }} g \in \mathbf {c}$ in terms of the arbitrary smooth function $\mathrm{\Upsilon }$ and *g* and its tensor invariants. (The formula is unwieldy and unenlightening, so we do not reproduce it here.) In the case of the metric $g_{\mathbf {E}} \in \mathbf {c}_{\mathbf {E}}$, computing gives, for example, that , which does not vanish for any $\mathrm{\Upsilon }$. Thus, $\mathbf {c}_{\mathbf {E}}$ admits no representative metric with respect to which the normal-form ambient metric is linear in $\rho$, and hence it cannot be conformally equivalent to $\mathbf {c}_{f, g}$ for any *f*, *g*.

### $\mathrm{G}_2^*$ holonomy

Here we show that the metric ${\smash {\widetilde{g}}}_{\mathbf {E}}$ produced in the previous subsection has holonomy equal to $\mathrm{G}_2^*$. We use the following theorem:

#### Theorem 5.3

([22, Theorem 1.1]) Let $\mathbf {D}$ be an oriented, real-analytic (2, 3, 5) distribution. Then, the metric holonomy  of any real-analytic ambient metric ${\smash {\widetilde{g}}}_{\mathbf {D}}$ for the conformal structure $\mathbf {c}_{\mathbf {D}}$ is contained in $\mathrm{G}_2^*$.

#### Proposition 5.4

The metric holonomy  of the ambient metric $({\smash {\widetilde{H}}}, {\smash {\widetilde{g}}}_{\mathbf {E}})$ is equal to $\mathrm{G}_2^*$.

#### Proof

By Theorem 5.3, , so to prove the claim, it suffices to show that the dimension of the holonomy group (or equivalently its Lie algebra) is equal to $\dim \mathrm{G}_2^* = 14$. (In fact, since the maximal subgroups of $\mathrm{G}_2^*$ are the maximal parabolic subgroups, all of which have dimension 9, it suffices to show that .)

By the Ambrose–Singer Theorem [4], the Lie algebra of  contains (in fact, since ${\smash {\widetilde{g}}}_{\mathbf {E}}$ is real-analytic, is equal to) the *infinitesimal holonomy algebra* of ${\smash {\widetilde{g}}}_{\mathbf {E}}$ at any point ${\smash {\widetilde{u}}} \in {\smash {\widetilde{H}}}$: This is the Lie algebra  generated by the value of its curvature ${\smash {\widetilde{R}}}$ and the derivatives thereof at ${\smash {\widetilde{u}}}$, or more precisely, the endomorphisms , where $X, Y, Z_1, \ldots , Z_k \in T_{\tilde{u}} {\smash {\widetilde{H}}}$ and ${\smash {\widetilde{\nabla }}}$ is the Levi-Civita connection of ${\smash {\widetilde{g}}}_{\mathbf {E}}$. Computing gives that the image of ${\smash {\widetilde{R}}}_{\tilde{u}}$ in  (where the basepoint ${\smash {\widetilde{u}}} \in {\smash {\widetilde{H}}}$, which we suppress below, is any point with $t = 1, \rho = 0$), is spanned by the linearly independent endomorphisms ${\smash {\widetilde{R}}}_{12}, {\smash {\widetilde{R}}}_{14}, {\smash {\widetilde{R}}}_{16}, {\smash {\widetilde{R}}}_{23}, {\smash {\widetilde{R}}}_{24}, {\smash {\widetilde{R}}}_{26}$, where  and . Those elements, together with , , , , ${\smash {\widetilde{\nabla }}}_{\partial _{\rho }} {\smash {\widetilde{R}}}_{12}$, , , ${\smash {\widetilde{\nabla }}}_{\partial _{\rho }} {\smash {\widetilde{R}}}_{16}$, comprise a basis of the space of endomorphisms generated by at most one derivative of ${\smash {\widetilde{R}}}$. But this basis has $\dim \mathrm{G}_2^* = 14$ elements. $\square$

Instead of using Theorem 5.3 one can alternatively show the containment  by verifying that (a) the 3-formis parallel with respect to ${\smash {\widetilde{\nabla }}}$ and (b) for any (equivalently, every) ${\smash {\widetilde{u}}} \in {\smash {\widetilde{H}}}$, the stabilizer of ${\smash {\widetilde{\mathrm{\Phi }}}}_{\tilde{u}}$ in $\mathrm{GL}(T_{\tilde{u}} {\smash {\widetilde{H}}})$ is isomorphic to $\mathrm{G}_2^*$ and contained in ; here,  and .

#### Remark 5.5

The result [22, Theorem 1.2] gives conditions on real-analytic, oriented (2, 3, 5) distributions $\mathbf {D}$ that are together sufficient to guarantee that any associated real-analytic ambient metric has holonomy $G_2^*$. Though the conditions hold for almost all such distributions (in a sense that can be made precise), they do not all hold for $\mathbf {E}$, and hence that result cannot be used to prove Proposition 5.4: One of the conditions is that there is a point *u* at which the fundamental curvature $A_u$ does not have a multiple root in , but as we found in the proof of Proposition 3.1 for $\mathbf {E}$ the fundamental curvature has a quadruple root at every point.

### Projective structure with normal projective holonomy $\mathrm{G}_2^*$

By [21] we may regard the real-analytic ambient manifold of a real-analytic conformal structure of odd dimension $n \geqslant 3$ and signature (*p*, *q*) as the so-called Thomas cone of a projective structure of dimension $n + 1$, which is defined on the space of orbits of the $\mathbb {R}_+$-action defined by the dilations $\delta _s$. This identifies the Levi-Civita connection of the ambient metric with the normal tractor connection—a natural, projectively invariant vector bundle connection on a particular vector bundle of rank $n + 2$ over and canonically associated to the projective manifold—whose holonomy is thus reduced to .

Thus, the metric ${\smash {\widetilde{g}}}_{\mathbf {E}}$ can be used to construct an explicit 6-dimensional projective structure  whose normal projective holonomy is equal to $\mathrm{G}_2^*$. Computing the connection forms of ${\smash {\widetilde{\nabla }}}$ and applying the formula in [21, Remark 6.6] (using the section ) gives an explicit representative connection $\nabla$ in $\mathbf {p}$; the connection is characterized by the covariant derivative formulae in the “Appendix”.

Example 7.1 of [21] also gives a 1-parameter family of nontrivial 6-dimensional projective structures with normal holonomy contained in $\mathrm{G}_2^*$, but for every member of that family the containment is proper.

## Appendix: Data for the connection in Sect. 5.2

Here we give the nonzero connection forms $\omega _{ab}^c$ of the connection $\nabla$ defined in Sect. 5.2 in the frame $(E_1, \ldots , E_6)$, where we set $E_6 = \partial _{\rho }$, on $H {\times } ({-}2, \infty )$. These are characterized by $\nabla _{E_a} E_b = \omega _{ab}^c E_c$.


## Footnotes

1 In fact, Cartan [13] and Engel [17] (separately) first discovered this relationship in simultaneous notes in 1893: They gave explicit distributions on $\mathbb {C}^5$ whose infinitesimal symmetry algebras are isomorphic to the complex simple Lie algebra $\mathfrak {g}_2^{\mathbb {C}}$, hence furnishing the first explicit realization of an exceptional such algebra. See [3] for a concise history of the topic.

2 Elsewhere (2, 3, 5) distributions are sometimes instead termed *generic* 2-plane distributions (on 5-manifolds) [12, 28].

3 Since $\mathrm{G}_2^*$ is semisimple and *Q* is parabolic, this geometry is an example of an important class of structures called *parabolic geometries*. For any such geometry one can encode any structure on a manifold *M* in a bundle $E \rightarrow M$ equipped with a canonically determined Cartan connection; see the standard reference [12] for a general treatment of parabolic geometries and Section 4.3.2 there for discussion of the geometry of (2, 3, 5) distributions in this setting. Robert Bryant has observed that the pseudoconnection Cartan produces is not in fact a Cartan connection [9].

4 Cf. the notion of Petrov type in four-dimensional Lorentzian geometry.

5 For any ambient metric in normal form with respect to a metric *g*, the linear term in the Taylor expansion of  about $\rho = 0$ is a particular linear combination of *g* and $\mathrm{Ric}^g$ [18, (3.6)], so it follows from (5) and (7) without further computation that this claim is true for the first derivative, $g_{ab}'(0)$.
